# A Full-Term Pregnancy in a Patient With Uterus Didelphys

**DOI:** 10.7759/cureus.66937

**Published:** 2024-08-15

**Authors:** Ahmed S Gaily, Nada A Abdulaal, Afaf Alzahrani

**Affiliations:** 1 Obstetrics and Gynaecology, Al-Kharj Military Industries Corporation Hospital (AKMICH), Al-Kharj, SAU; 2 Obstetrics and Gynaecology, Al Kharj Armed Forces Hospital, Al-Kharj, SAU

**Keywords:** mullarian fusion anomaly, transverse vaginal septum, two cervix, full-term pregnancy, uterine didelphys, mullarian duct anomalies

## Abstract

Müllerian duct anomalies (MDAs) are congenital uterine abnormalities resulting from incorrectly developed Müllerian ducts. Uterus didelphys is an uncommon MDA linked to fetal growth restriction, dysmenorrhea, dyspareunia, and higher rates of infertility. We present an unusual case of a 21-year-old woman who presented at the emergency room with no previous history of medical illness. An ultrasound report showed her uterus with an irregular shape. An official departmental scan indicated an anteverted, bicornuate uterus harboring a normal-looking intrauterine gestational sac and a 2.5 cm cervical length. Because of this borderline cervical length and the associated anomaly, the patient was scheduled for an elective cervical cerclage. Before starting the procedure, the patient was found to have a thick longitudinal vaginal septum and the cervix could not be evaluated for which the procedure was halted and the patient kept on progesterone. Elective cesarean surgery (LSCS, lower-section cesarean surgery) at 37 weeks of gestation was arranged due to the previous findings and breech presentation. During the LSCS, the examination revealed the presence of two non-communicating uteri, two cervical canals, and two separate vaginas dividing the introitus into two distinct openings. Diagnostic modalities such as magnetic resonance imaging (MRI), hysteroscopy, laparoscopy, hysterosalpingogram, and ultrasound (USG) are necessary for precise diagnosis of uterine didelphys. This case highlights that with careful prenatal and intrapartum supervision good pregnancy outcomes can be achieved in a uterine didelphic condition.

## Introduction

Congenital uterine abnormalities that arise from the improper development of the Müllerian ducts are known as Müllerian duct anomalies (MDAs). The incidence of MDAs in the general population is estimated to be between 3% and 5%. During embryonic development, the mesonephric (Mullerian) ducts can fuse abnormally, resulting in uterine anomalies, such as septate, didelphic, unicornuate, and bicornuate uteri. The type of uterine anomaly affects fertility and the course of pregnancy [1].

These abnormalities can present with a variety of symptoms, ranging from asymptomatic cases to amenorrhea, dyspareunia, dysmenorrhea, persistent pelvic discomfort, pregnancy loss, fetal malpresentation, placental abruption, and intrauterine growth restriction [2].

In patients with a history of miscarriages and malpresentation, considering pregnancies that develop in a malformed uterus is crucial, as they are common and often asymptomatic. Diagnostic tools such as magnetic resonance imaging (MRI), hysteroscopy, laparoscopy, hysterosalpingogram, and ultrasound sonography (USG) are essential for accurate diagnosis [3].

While studies show that this condition may be associated with various obstetrical complications, from preterm labor to severe outcomes, including uterine rupture, many studies report successful deliveries in patients with MDAs [4].

## Case presentation

A 21-year-old woman with no prior medical history was referred from a private hospital to our emergency department owing to a US report indicating an abnormal uterine shape. She was a primigravida with a spontaneous conception at five weeks gestation.

A departmental scan conducted two weeks later revealed an anteverted bicornuate uterus. The right endometrial cavity contained a single intrauterine gestational sac with a visible embryo, having a crown-rump length corresponding to eight weeks and five days gestation, along with a subchorionic hematoma measuring 1.8 x 1.4 cm. The left endometrial cavity was empty, with a thickness of 2 cm and a heterogenous appearance. The right ovary contained a simple cyst measuring 2 x 2.8 cm, while the left ovary appeared normal.

The patient was started on progesterone and scheduled for antenatal clinic appointments. A follow-up scan at 13 weeks and six days gestation showed similar findings, with the growth of an embryo corresponding to the gestational age. In addition, the previously observed subchorionic hematoma had resolved, and the cervical length was 2.5 cm.

The medical team discussed the findings and management plan with the patient. Owing to the borderline cervical length and associated Müllerian anomaly, which placed the patient at high risk for second-trimester miscarriage and preterm labor, the team decided on prophylactic cervical cerclage. After thorough counseling on the benefits, risks, and potential complications, the patient was admitted for the procedure at 14 weeks and three days gestation. The patient understood, accepted, and provided consent.

**Figure 1:**
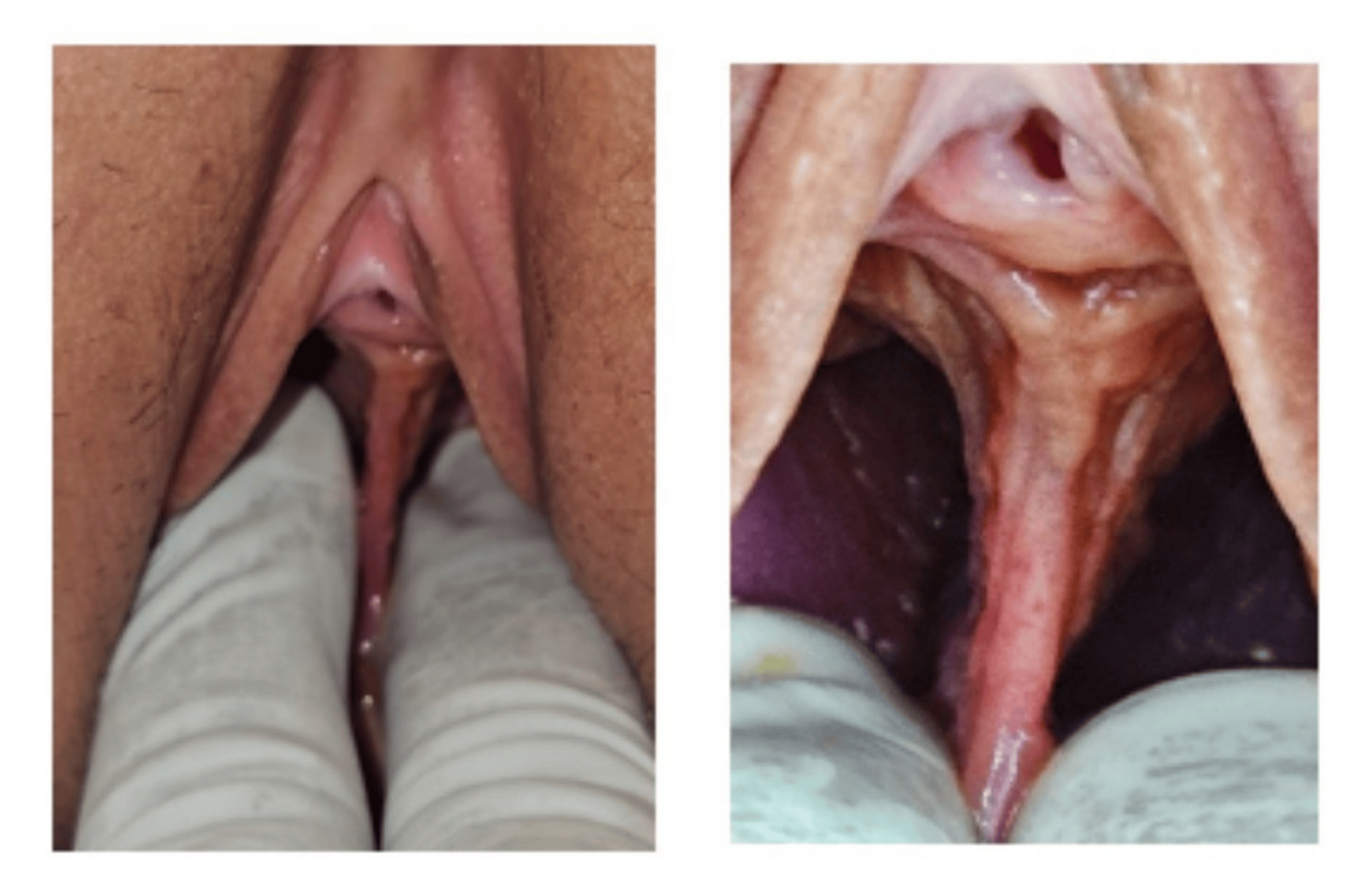
Longitudinal vaginal septum dividing full vaginal length into two canals.

Under spinal anesthesia, the patient was positioned in a dorsal lithotomy, cleaned, and draped. An examination under anesthesia revealed a thick longitudinal, non-obstructing vaginal septum (Figure 1) extending from the posterior aspect of the urethral meatus to the fourchette, creating two vaginal cavities. To minimize interference with the gravid uterus, the number of cervixes was not determined.

The patient commenced treatment with vaginal progesterone and underwent a kidney, ureter, and bladder scan, which revealed a normal urinary tract without significant abnormalities. She attended regular antenatal follow-up appointments. The results of an anomaly scan at 22 weeks gestation showed a single viable breech fetus with parameters and weight corresponding to gestational age, posterior placenta situated away from the cervix, and normal amniotic fluid levels. No fetal anomalies were detected at the time of the scan. Serial growth scans remained within normal limits, and the pregnancy progressed smoothly. The patient was booked for an elective cesarean section at 37 weeks gestation. She was also administered steroids to enhance fetal lung maturity.

**Figure 2:**
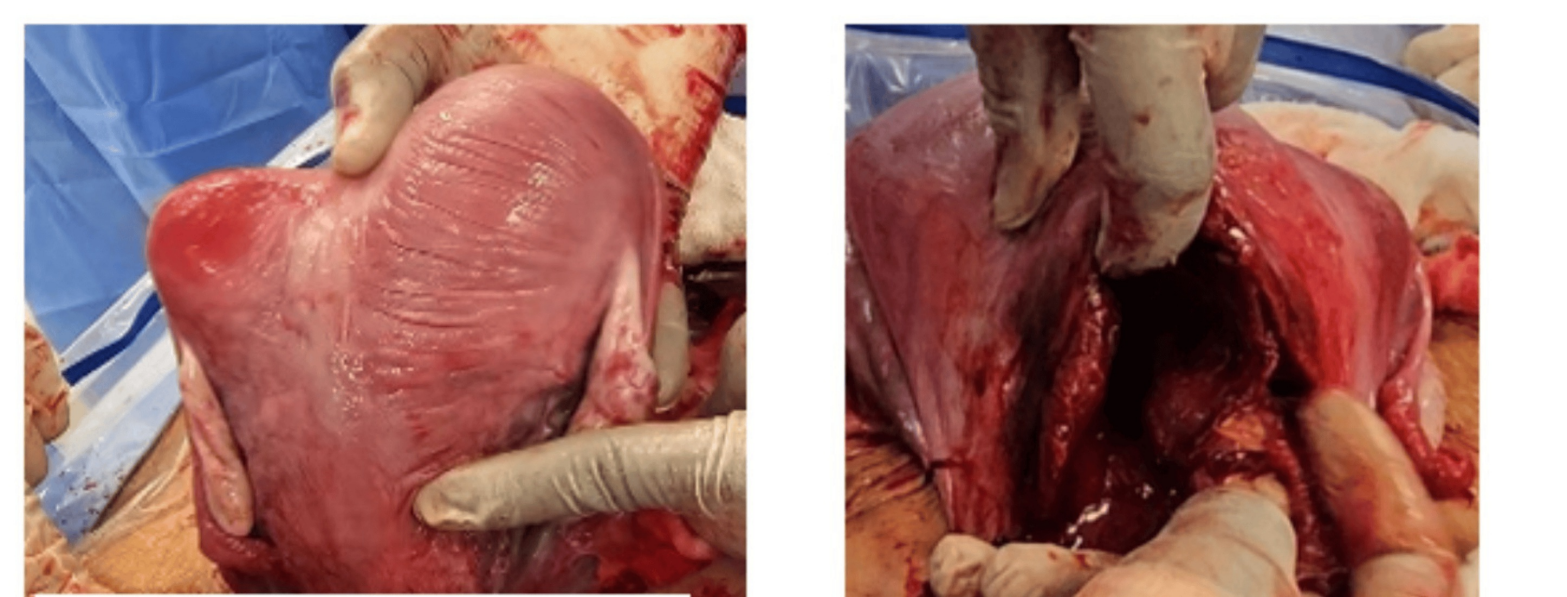
Intraoperative findings of uterine didelphys, left picture showing two endometrial cavities, first indicated by the surgeon’s left index finger and second by the right thumb.

**Figure 3:**
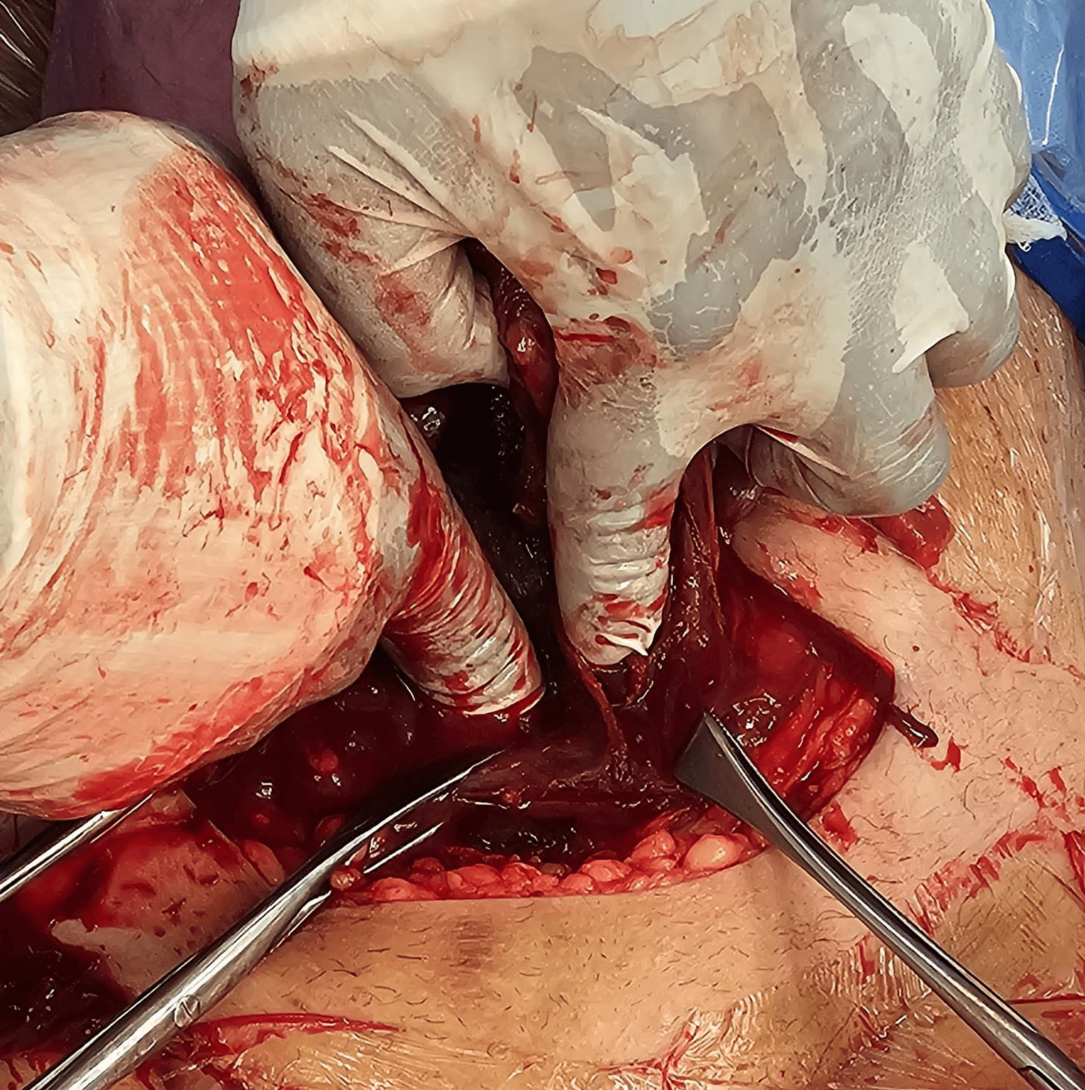
Intraoperative finding of two cervical canals, marked by the surgeon's fingers

During the operation, a sterilization protocol was strictly followed. A Pfannenstiel incision was made, and the anterior abdominal wall was opened in layers. A small transverse incision was created in the lower uterine segment, through which a healthy 2.9-kg baby boy with APGAR scores of 7 and 9 was delivered, along with the placenta. The uterus was then exteriorized for proper examination. Upon inspection, we found two incised uteri (Figure 2). The right uterine cavity contained the baby, while the other was examined. An endometrial biopsy was sent for histopathology, which revealed a normal residual reaction. Two cervical canals were found as well (Figure 3). Each uterine incision was sutured separately in the standard two-layer fashion.

The two ovaries and fallopian tubes appeared normal. A speculum examination at the end of the procedure revealed two cervical canals and two vaginas, each with a separate introitus. The estimated blood loss was 500 mL.

The final classification was recorded as U3C2V1 according to the European Society of Human Reproduction and Embryology/European Society for Gynaecological Endoscopy (ESHRE/ESGE) guidelines [5]. The postoperative hospital stay was uneventful, and the patient was discharged in stable condition. No puerperal complications were observed, and the patient attended her postnatal follow-up visit six weeks later in good health with no complaints.

## Discussion

Uterus didelphys is one of the rarest MDAs. This condition is associated with fetal growth restriction, dysmenorrhea, dyspareunia, and higher rates of infertility. Owing to its rarity, data on the overall results of this congenital anomaly are limited. However, this may be linked to several pregnancy-related and non-pregnancy-related issues. Uterine didelphys is correlated with increased rates of infertility [4], 30% preterm delivery rate, and a miscarriage incidence of approximately 33%. Diagnosis is commonly conducted through US monitoring [6], with two-dimensional US being the primary modality for assessing the uterus and adnexa [7]. Pregnant women with MDAs should be closely monitored obstetrically during their pregnancies and be informed about the higher incidence of pregnancy-related problems [4].

Fusion anomalies result in didelphic uteri. According to the American Society for Reproductive Medicine, a bicornuate uterus is characterized by an exterior fundal depression larger than 1 cm without specifying the degree of myometrial fusion. By contrast, the uterus didelphys consists of two distinct uterine bodies with duplicated cervices. The three variants of uterus didelphys are (1) uterus didelphys with a complete longitudinal vaginal septum, (2) uterus didelphys with or without a longitudinal vaginal septum of variable length, and (3) uterus didelphys with an obstructed R/L hemi-vagina [3]. This case is particularly significant, as the pregnancy was spontaneous and reached full term without complications, despite the patient having uterus didelphys and a longitudinal vaginal septum.

The first-line modalities for assessing the female reproductive tract, including MDAs involving the uterus, cervix, and vagina, are two-dimensional transabdominal US and endovaginal US [3]. Uterus didelphys, a rare MDA, accounts for approximately 5% of cases. Early detection can alleviate patient concerns and improve pregnancy outcomes. However, obstetricians must educate patients about the potential consequences of this condition. At 37 weeks of gestation, with a breech presentation, the medical team decided on an elective C-section to mitigate potential obstetrical complications. For singleton breech pregnancies at or after 39 weeks, most guidelines recommend a cesarean section as the preferred course of action. The Term Breech Trial in 2000 and Premoda in 2006 are two significant studies that offered clinical support for cesarean sections [8].

Histological examination of one uterus showed an endometrium with a decidual response. Furthermore, the presence of two distinct introituses in each of the two vaginas and two cervical canals underscores the limitations of the imaging modalities such as the US. Patients with uterus didelphys resulting from a Müllerian defect do not typically experience difficulties conceiving, but they are at higher risk for premature labor, malpresentation, and malposition [9].

This case report demonstrates that good pregnancy outcomes can be achieved with careful prenatal and intrapartum supervision in cases of uterus didelphys. This report also emphasizes that while uterus didelphys is a rare condition, emergency physicians should be aware of MDAs owing to their association with higher rates of infertility and pregnancy complications [4].

## Conclusions

Uterus didelphys, a type of Müllerian malformation resulting from improper fusion of uterine ducts, can be identified before or during pregnancy. In our case, uterus didelphys was partially detected prenatally through US imaging. While US monitoring is commonly used to diagnose this condition, this line of investigative modalities has its limitations. Uterus didelphys may not decrease fertility; however, it can lead to undesirable pregnancy outcomes, such as preterm labor, malpresentation, malposition, and/or uterine rupture. Most guidelines recommend a cesarean section for singleton breech pregnancies at a gestational age of at least 39 weeks. A key conclusion is that uterus didelphys can carry a pregnancy to term, but obstetricians must educate patients about potential complications. This case further emphasizes that, while MDAs are uncommon, early detection can alleviate patient concerns and yield better pregnancy outcomes.
